# Impact of dexamethasone, etoposide, ifosfamide and carboplatin as concurrent chemoradiotherapy agents for nasal natural killer/T-cell lymphoma

**DOI:** 10.3892/mco.2013.123

**Published:** 2013-05-17

**Authors:** YOSHIOMI HATAYAMA, MASAHIKO AOKI, HIDEO KAWAGUCHI, YUICHIRO NARITA, KATSUMI HIROSE, MARIKO SATO, YOSHIHIRO TAKAI

**Affiliations:** Department of Radiology, Hirosaki University School of Medicine, Hirosaki, Aomori 036-8562, Japan

**Keywords:** carboplatin, chemoradiotherapy, dexamethasone, etoposide, extranodal natural killer/T-cell lymphoma, ifosfamide

## Abstract

The nasal type of extranodal natural killer (NK)/T-cell lymphoma (NKTCL) is a rare aggressive lymphoma with poor prognosis. The reported 5-year overall survival for patients with localized nasal NKTCL treated with cyclophosphamide, hydroxydaunorubicin, oncovin and prednisone (CHOP) is <50%. Dexamethasone, etoposide, ifosfamide and carboplatin (DeVIC) chemotherapy was designed as a salvage chemotherapeutic regimen for aggressive lymphoma, comprising multidrug resistance (MDR) non-related agents and etoposide, which is considered to be effective against nasal NKTCL. An experimental chemoradiotherapy (CRT) is currently being designed using DeVIC as the concurrent chemotherapeutic regimen for nasal NKTCL. The aim of this study was to examine the initial outcome of this treatment and evaluate its effectiveness and feasibility. Six patients (age range, 29–82 years; median age, 68 years) were treated with CRT using DeVIC between April, 2004 and February, 2010. The median follow-up was 56 months (range, 11–80 months). All patients were administered 3–6 cycles of full-dose DeVIC regimen. The chemotherapy was administered concurrently with radiotherapy (RT) and was repeated every 3 weeks. RT was performed using 4-MV X-ray and the prescription dose was 46–50 Gy/23–25 fx (median, 50 Gy). After treatment, all patients were followed up at our hospital. A complete remission was achieved in 5 patients (83%) at 1 month after treatment. The 5-year overall survival and disease-free survival rates were 100%. No severe adverse effect (grade ≥3) was reported. In conclusion, the initial results of the experimental CRT with DeVIC for this type of aggressive lymphoma were very encouraging. Further investigation is required on concurrent CRT with 50 Gy/25 fx and 3 cycles of DeVIC comprising non-MDR agents and etoposide for nasal NKTCL.

## Introduction

The nasal type of extranodal natural killer (NK)/T-cell lymphoma (NKTCL) is a rare aggressive lymphoma with a poor prognosis and is more commonly encountered in East Asia (1). Nasal NKTCL refers to tumors arising in the nose, paranasal sinuses and nasopharynx (2,3). It accounts for 3% of malignant lymphomas in Japan and it is an Epstein-Barr virus-associated lymphoma (4–6). The lymphoma cells express P-glycoprotein, which results in multidrug resistance (MDR) of the tumors (7–10). The disease primarily affects middle-aged males. Nasal bleeding, nasal congestion, rhinitis and facial swelling are the predominant symptoms of this disease. Treatment approaches include radiotherapy (RT), chemotherapy, or a combination of the two. Complete remission was observed in ∼72–78% of patients with RT alone; however, 50–60% of these cases eventually relapsed (11–15). The reported 5-year overall survival rate for patients with localized nasal NKTCL treated with cyclophosphamide, hydroxydaunorubicin, oncovin and prednisone (CHOP) was <50% (16–18). Treatment results of stage I–II disease have recently improved; however, a standard treatment has yet to be established. Dexamethasone, etoposide, ifosfamide and carboplatin (DeVIC) chemotherapy, which was designed as a salvage chemotherapeutic regimen for aggressive lymphomas, comprises non-MDR agents and etoposide and is considered to be effective against nasal NKTCL (19,20). Currently, we are experimenting with chemoradiotherapy (CRT) with DeVIC as the concurrent chemotherapeutic agents for nasal NKTCL. The present study aimed to evaluate the initial outcomes with this treatment and to assess its effectiveness and feasibility.

## Materials and methods

### General

Six patients with nasal NKTCL underwent CRT at Hirosaki University Hospital (Hirosaki, Japan) and Aomori Prefectural Hospital (Aomori, Japan) between April, 2004 and February, 2010. The median follow-up time was 56 months (range, 11–80 months). All 6 patients were staged according to the Ann Arbor staging criteria. Complete patient evaluation included physical examination, blood counts, screening blood chemistry tests (such as hepatic and renal function and lactate dehydrogenase tests), chest radiographs, whole-body computed tomography (CT) and/or positron emission tomography (PET)-CT scans and bone marrow biopsies.

### Patient characteristics

The study included 4 males and 2 females with a median age of 68 years (age range, 29–82 years). In all 6 patients, the primary tumor originated in the nasal cavity. In 5 patients, the tumor involved the nasal cavity unilaterally and in 1 patient it involved the nasal cavities bilaterally and the paranasal sinuses. Five patients had stage IE disease and 1 patient had stage IIE disease. The most frequent presenting symptom was nasal congestion, which was observed in 5 patients. B symptoms were not observed in any of the patients. Elevated lactate dehydrogenase levels were observed in 3 patients. The ratio for performance status (PS) was 0:1 = 3:3 and for International Prognostic Index score was low:low-intermediate = 3:3 (Table I).

### Chemotherapy

The patients underwent 3–6 cycles of full-dose DeVIC regimen. The drug doses and administration schedule were as follows: dexamethasone (40 mg/day on days 1–3), etoposide (100 mg/m^2^ on days 1–3), ifosfamide (1.5 mg/m^2^ on days 1–3) and carboplatin (300 mg/m^2^ on day 1). The chemotherapy was administered concurrently with RT and was repeated every 3 weeks (Table II).

### Radiotherapy

**Figure 1:**
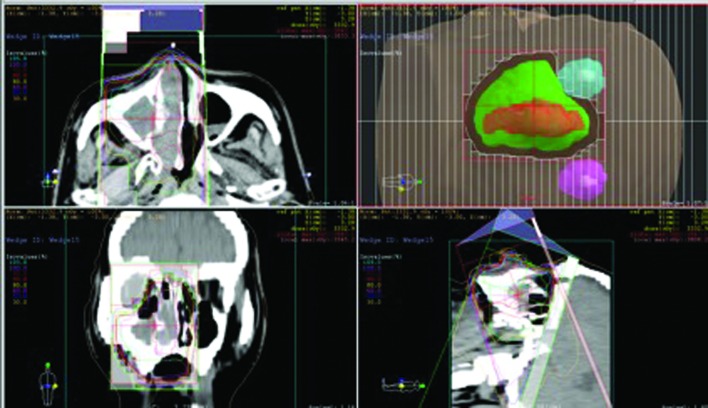
Three-dimensional dose distributions for a patient who received a total dose of 50 Gy with two fixed non-coplanar conformal beams. Volume: red, GTV; green, CTV.

The patients received RT from a linear accelerator using 4-MV X-rays. The clinical target volume included the gross tumor volume and the entire nasal cavity and ipsilateral paranasal sinus. The patients underwent RT with a conventional fractionation schedule at a median dose of 50 Gy (range, 46–50 Gy) (Table II). A three-dimensional dose distribution for a patient with a large tumor is shown in Fig. 1.

## Results

The present study included 4 males and 2 females with a median age of 68 years (age range, 29–82 years) who were treated with CTR using DeVIC in order to assess its effectiveness. Following treatment, the patients were followed up for a median of 56 months (range, 11–80 months).

**Figure 2:**
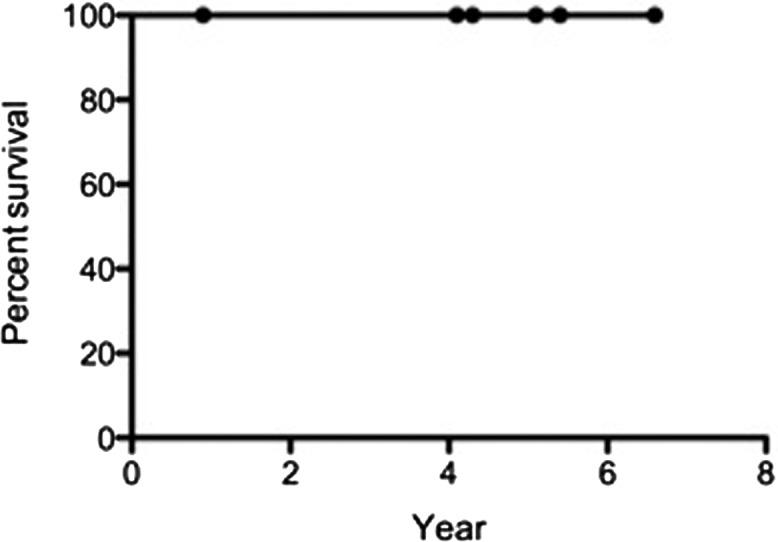
Overall survival.

**Figure 3:**
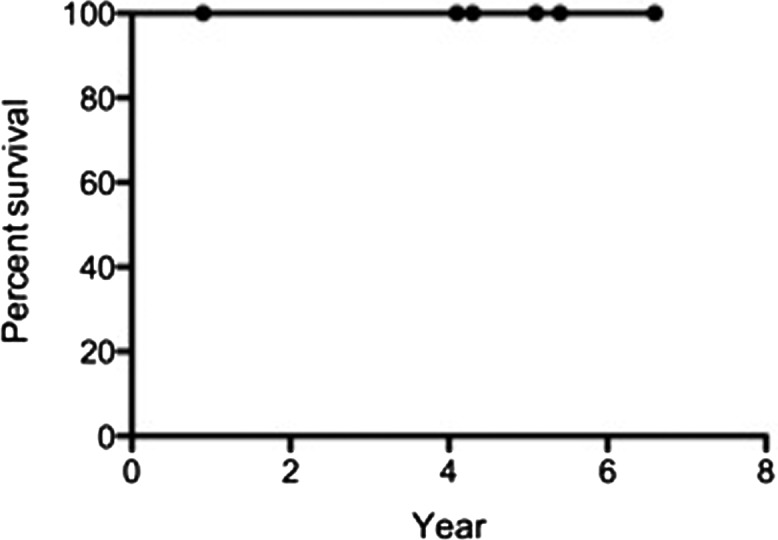
Disease-free survival.

**Figure 4:**
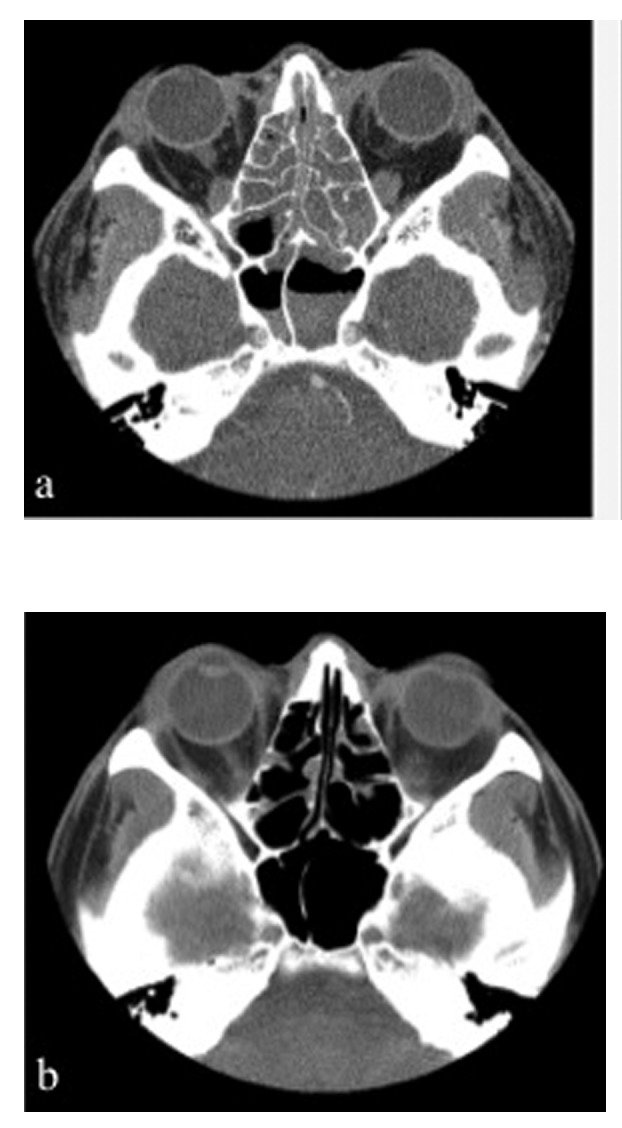
A 29-year-old male received a total dose of 50 Gy and 6 cycles of dexamethasone, etoposide, ifosfamide and carboplatin (DeVIC). (a) Computed tomography (CT) scan prior to chemoradiotherapy (CRT) shows a naso-paranasal space-occupying soft tissue mass. (b) The soft tissue mass completely disappeared 1 month after the completion of CRT.

**Figure 5:**
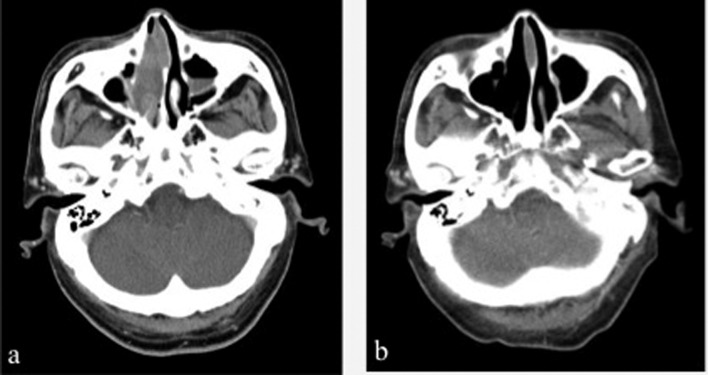
A 72-year-old male received a total dose of 50 Gy and 4 cycles of dexamethasone, etoposide, ifosfamide and carboplatin (DeVIC). (a) Computed tomography (CT) scan prior to chemoradiotherapy (CRT) shows a right nasal space-occupying soft tissue mass. (b) The mass completely disappeared 1 month after the completion of CRT.

Complete remission was achieved in 5 patients (83%) after 1 month of treatment. The 5-year overall survival and disease-free survival rates were 100% (Figs. 2 and 3). Although grade 1–2 radiation-induced mucositis of the nasal and oral cavities was observed in 3 patients, no severe adverse effects (grade 3) have yet been reported in any of the patients. Two representative cases are shown in Figs. 4 and 5. CT scans prior to CRT reveal a naso-paranasal and a right nasal space-occupying mass of soft tissue. However, 1 month following the completion of CRT, the mass of soft tissue completely disappeared.

## Discussion

Following treatment with DeVIC and CRT, the results showed the complete remission rate to be 83%, which was higher compared to that reported by previous studies on chemotherapy followed by RT. Sakata *et al* reported that radiation doses >52 Gy may be required to obtain local control in patients with localized nasal NKTCL (21). Koom *et al* reported that radiation doses <45 Gy were significantly associated with local relapse (12). These studies have been cited by several textbooks. Our median radiation dose was 50 Gy (range, 46–50 Gy). However, Kim *et al* reported satisfactory local control with lower doses of ∼40 Gy (22).

DeVIC was added to the regimen for management of systemic relapse and consolidation. This regimen has been used in combination with RT since the late 1990s in Japan (20). All the patients received 3–6 cycles of a full-dose DeVIC regimen and 46–50 Gy of RT.

Severe mucositis and moderate dermatitis are the side effects of CRT in the nasal cavity; therefore, nutritional management and pain control are critical. In addition, the radiation dose to the optic nerve, optic chiasm and eyeball must be considered in order to avoid visual disorders. All patients completed the treatment without severe adverse events. To date, no visual disorders have been reported.

In previous retrospective studies, the local control and systemic relapse rates of nasal NKTCL were 18.9–47% and 15.1–81%, respectively (12,13,23,24). In those studies, the patients were either treated with RT alone or a combination of RT and CHOP-based chemotherapy. The 5-year survival rate for localized nasal NKTCL is 47–57% (17,18).

Thus far no local recurrence or systemic relapse has been observed in any of the patients. All 6 patients were still alive at the time of writing this manuscript. Our initial results from the present experimental CRT/DeVIC regimen for this aggressive type of lymphoma were encouraging. Additional investigation is required on concurrent CRT with 50 Gy/25 fx and 3–6 cycles of DeVIC comprising non-MDR agents and etoposide for nasal NKTCL.

Our study included patients who were under a short period of observation and our results suggest that the use of CRT with DeVIC is effective for the treatment of nasal NKTCL.

## Figures and Tables

**Table I:** Patient characteristics.

Variables	Value
Age (years)	
Range	29–82
Median	68
Gender	
Male	4
Female	2
Primary site	
Unilateral nasal cavity	5
Bilateral nasal cavities	1
Stage	
IE	5
IIE	1
B symptoms	
Yes	0
No	6
Lactate dehydrogenase elevation	
Yes	3
No	3
PS	
0	3
1	3
IPI score	
Low	3
Low-intermediate	3

PS, performance status; IPI, International Prognostic Index.

**Table II:** Treatment characteristics.

Variables	No. of patients
Radiation dose (Gy)	
50	4
46	2
Non-opposing pair	2
Multiple field	4
Chemotherapy (cycles)	
3	3
4	2
6	1
